# Sequential genomic analysis using a multisample/multiplatform approach to better define rhabdomyosarcoma progression and relapse

**DOI:** 10.1038/s41698-023-00445-1

**Published:** 2023-09-20

**Authors:** Henry de Traux de Wardin, Josephine K. Dermawan, Marie-Sophie Merlin, Leonard H. Wexler, Daniel Orbach, Fabio Vanoli, Gudrun Schleiermacher, Birgit Geoerger, Stelly Ballet, Delphine Guillemot, Eléonore Frouin, Stacy Cyrille, Olivier Delattre, Gaelle Pierron, Cristina R. Antonescu

**Affiliations:** 1 Department of Pathology and Laboratory Medicine, Memorial Sloan Kettering Cancer Center, New York, NY USA; 2 Unit of Somatic Genetics, Institut Curie, Paris, France; 3 University of Lorraine, Centre Hospitalier Régional Universitaire (CHRU), Childrens’ Hospital, Department of Pediatric Oncology, Vandoeuvre-lès-Nancy, France; 4 Department of Pediatrics, Memorial Sloan Kettering Cancer Center, New York, NY USA; 5 SIREDO Oncology Center (Care, Innovation and Research for Children, Adolescents and Young Adults with Cancer), PSL University, Institut Curie, Paris, France; 6 U830 INSERM, Paris, France; 7 Gustave Roussy Cancer Center, Department of Pediatric and Adolescent Oncology, Institut National de la Santé Et de la Recherche Médicale (INSERM) U1015, Université Paris-Saclay, Villejuif, 94805 France; 8 Department of Biometrics, Institut Curie, Paris, France

**Keywords:** Predictive markers, Oncogenesis, Paediatric cancer, Predictive markers

## Abstract

The genomic spectrum of rhabdomyosarcoma (RMS) progression from primary to relapse is not fully understood. In this pilot study, we explore the sensitivity of various targeted and whole-genome NGS platforms in order to assess the best genomic approach of using liquid biopsy in future prospective clinical trials. Moreover, we investigate 35 paired primary/relapsed RMS from two contributing institutions, 18 fusion-positive (FP-RMS) and 17 fusion-negative RMS (FN-RMS) by either targeted DNA or whole exome sequencing (WES). In 10 cases, circulating tumor DNA (ctDNA) from multiple timepoints through clinical care and progression was analyzed for feasibility of liquid biopsy in monitoring treatment response/relapse. ctDNA alterations were evaluated using a targeted 36-gene custom RMS panel at high coverage for single-nucleotide variation and fusion detection, and a shallow whole-genome sequencing for copy number variation. FP-RMS have a stable genome with relapse, with common secondary alterations *CDKN2A/B*, *MYCN,* and *CDK4* present at diagnosis and impacting survival. FP-RMS lacking major secondary events at baseline acquire recurrent *MYCN* and *AKT1* alterations. FN-RMS acquire a higher number of new alterations, most commonly *SMARCA2* missense mutations. ctDNA analyses detect pathognomonic variants in all RMS patients within our collection at diagnosis, regardless of type of alterations, and confirmed at relapse in 86% of FP-RMS and 100% FN-RMS. Moreover, a higher number of fusion reads is detected with increased disease burden and at relapse in patients following a fatal outcome. These results underscore patterns of tumor progression and provide rationale for using liquid biopsy to monitor treatment response.

## Introduction

Rhabdomyosarcomas (RMS) comprise a heterogeneous clinical and molecular group of high-grade sarcomas showing various degrees of myogenic differentiation. Despite significant progress in refining multimodality therapies, the survival of patients developing metastatic or relapsed disease is dismal^1–3^. This shortcoming also relates to the limited understanding of the genomic landscape evolution driving the metastatic progression or therapy relapse in RMS. Most previous genomic studies in RMS have focused on identification of molecular markers of prognostic impact, allowing for improved risk stratification and therefore guiding therapy intensity. Thus the presence of *PAX3/7::FOXO1* fusion and *MYOD1*-L122R mutation, indicative of alveolar or fusion-positive (FP-RMS), and spindle/sclerosing RMS histologies, respectively, trigger risk escalation. Additional genetic alterations, such as somatic *TP53* mutations, have been found to correlate with worse survival in both FP and FN RMS^4–6^, while the prognostic role of recurrent *CDK4* and *MYCN* amplifications detected mainly in FP-RMS^6,7^ remains to be further validated.

Given the increasing significance of molecular markers in RMS for both risk stratification and disease monitoring, it has become essential to adopt innovative approaches for their screening. Thus, recent technological advances have enabled genomic testing of circulating tumor-derived material released by tumor cells in the blood. The minimally invasive ‘liquid biopsies’ have many advantages, including overcoming the tumor heterogeneity and spatial limitations associated with tissue biopsies, as well as allow serial collection at multiple timepoints throughout the patient treatment and follow-up. As such, future applications of liquid biopsy may inform of real-time disease burden and treatment response, which will potentially change the approach in diagnosis, risk stratification, and monitoring. Moreover, large studies investigating the sensitivity of different RNA- or DNA-based NGS panels across various samples types (blood, bone marrow, etc.) have not been systematically conducted in RMS, in particular to assess the circulating tumor DNA (ctDNA) dynamics during patient therapy.

In this study we investigate genomic patterns of tumor progression in a well-annotated clinical and molecular cohort of FP and FN RMS, showing that FP-RMS exhibit relatively stable genomes at relapse, while secondary alterations present at diagnosis, i.e., *CDKN2A/B*, *MYCN*, and *CDK4*, impacted survival. Moreover, we explore the sensitivity of various deep-targeted and shallow whole-genome NGS platforms to define the best genomic approach of using liquid biopsy in future prospective clinical trials. Thus, ctDNA analysis identifies diagnostic variants in most RMS patients, at presentation and progression, with higher fusion reads correlating with disease burden and relapse.

## Results

### Patient population

A total of 35 patients with relapsed and metastatic RMS (18 FP-RMS, 17 FN-RMS) were included in the analysis (20 MSKCC, 15 Institut Curie). Clinical and tumor characteristics are summarized in Table 1. Most were <21 years (33/35), with 46% of patients ≥10 years. The primary location distribution was 37% in the extremity, 31% head and neck, 20% abdominopelvic, 6% para-meningeal, and 6% thorax. All cases were treated with multimodal chemotherapy comprised of vincristine, ifosfamide, and actinomycine-D. Some cases depending on the trial in which they were included additionally received etoposide, irinotecan, cyclophosphamide, or doxorubicine.

**Table 1:** Patient demographics and tumor characteristics grouped by originating institution.

Variable	Curie, *N* = 15	MSK, *N* = 20	Total, *N* = 35
Age group^a^			
Pediatric	10 (66.7%)	9 (45.0%)	19 (54.3%)
Adolescent/Young adult	5 (33.3%)	11 (55.0%)	16 (45.7%)
Sex			
Female	9 (60.0%)	9 (45.0%)	18 (51.4%)
Male	6 (40.0%)	11 (55.0%)	17 (48.6%)
Site			
Head and neck	5 (33.3%)	8 (40.0%)	13 (37.1%)
Abdominopelvis	3 (20.0%)	4 (20.0%)	7 (20.0%)
Extremity	7 (46.7%)	6 (30.0%)	13 (37.1%)
Thorax	0 (0.0%)	2 (10.0%)	2 (5.7%)
Histology/Fusion			
Fusion-Positive RMS	8 (53.3%)	10 (50.0%)	18 (51.4%)
Fusion-Negative RMS	7 (46.7%)	10 (50.0%)	17 (48.6%)
Size			
≤5 cm	7 (47.0%)	4 (20.0%)	11 (31%)
>5 cm	8 (53.0%)	16 (80.0%)	24 (69%)
N (TNM Classification)			
0	5 (33%)	11 (55%)	16 (46%)
1	10 (77%)	9 (45%)	19 (54%)
M (TNM Classification)			
0	9 (60%)	15 (75.0%)	24 (69%)
1	6 (40%)	5 (25.0%)	11 (31%)
RMS Risk group (COG)			
High	13 (87%)	12 (60.0%)	25 (71%)
Intermediate	2 (13%)	7 (35.0%)	9 (26%)
Low	0 (0.0%)	1 (5.0%)	1 (3%)
Relapse site			
Local	6 (42.9%)	9 (47.4%)	15 (45.5%)
Metastatic	8 (57.1%)	7 (36.8%)	15 (45.5%)
Regional	0 (0.0%)	3 (15.8%)	3 (9.1%)
NA	1	1	2
5-year Overall Survival rates [95% IC]	31% [13–73]	35% [13–89]	33% [18–62]

^a^Pediatric: 0–11 years; adolescent/young adult >12 years.

The MSK cohort included 10 FP-RMS and 10 FN-RMS. The median age was 14 years (range 0.6–24 years) for FP-RMS and 7.8 years (range 1.6–54 years) for FN-RMS. All samples were studied on MSK-IMPACT targeted DNA-based gene sequencing. For each patient at least two samples were investigated, most having paired primary and metastatic/relapsed tumor. Sixteen patients had one primary and one relapse sample analyzed, and remaining 4 (2 FN-RMS, 2 FP-RMS) had two different relapse samples tested. In FP-RMS, the *FOXO1* fusion was evaluated by Archer FusionPlex which showed *PAX3::FOXO1* fusion in 8 cases and *PAX7::FOXO1* fusion in 2.

The 15 patients from Institut Curie (IC) (8 FP-RMS, 7 FN-RMS) had a median age of 8.4 years (range 0.1–22 years) and 4.0 years (range 0.02–14 years), respectively. All cases with FP-RMS tumors showed a *PAX3::FOXO1* fusion by RNAseq. The genomic landscape of primary and relapse tumors was determined by either WES (*n* = 11) or combined targeted DNA (Dragon panel) and WES (*n* = 4), with targeted sequencing on primary and WES on relapse.

Within the entire cohort, the median time from diagnosis to relapse was 1.3 years (range 0.2–4.9 years), with 40% metastatic and 60% loco-regional relapse. Most had high-risk features at diagnosis (71%), such as metastatic disease (31%), nodal involvement (54%), large tumors (69%), and unfavorable location (extremity or parameningeal, 37%). The remaining were intermediate risk (26%) and one patient was defined as low risk. All patients were treated following established protocol guidelines except one lacking treatment data and therefore excluded from survival analysis. None of the above variables were significantly different among the two cohorts, including similar OS and EFS by Kaplan–Meier curves. Overall the 5-year OS was 33%.

### Primary and metastatic/relapsed tumor samples—sequential genomic analysis

This analysis was conducted to evaluate the acquired mutational landscape upon drug failure and metastasis in RMS, to better define genomic outlines of relapse that can be monitored through liquid biopsy.

**Fig. 1:**
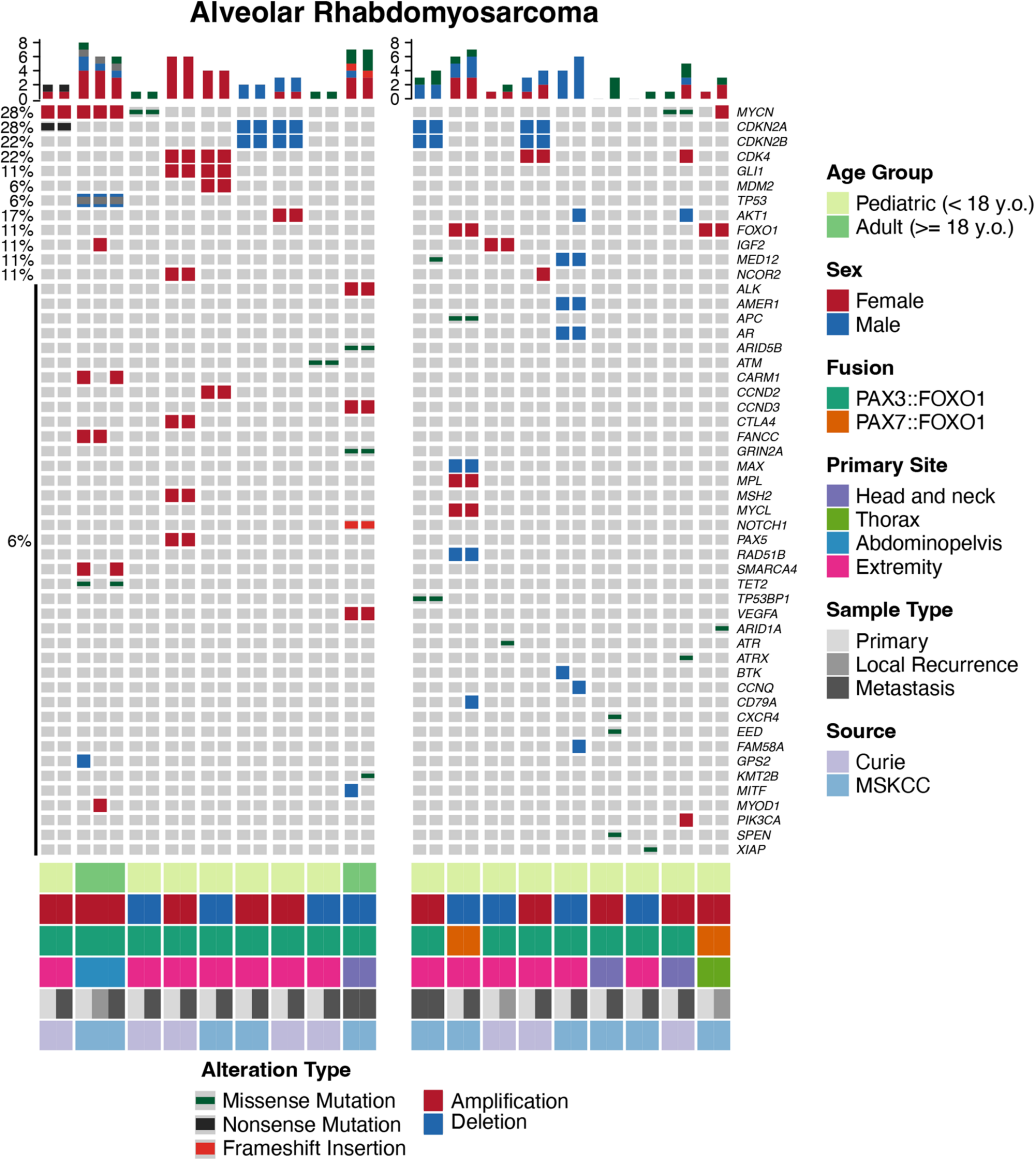
Oncoprint representation of SNV or CNV alterations in the entire FP-RMS cohort (37 samples, 18 patients). Each sample represents a column and each gene query is listed in a row. Samples from the same patients are grouped together and color-coded (shades of gray) based on primary tumor, local recurrence, or distant metastasis. Institution, age, sex, fusion type, and tumor site are color-coded. Frequency of gene alterations (left column, %) is applied to the whole sample cohort per patient (*n* = 37 tumors; 18 patients). Left side plot groups the patients lacking a clonal evolution with relapse, while right side plot depicts patients who acquired new major secondary alterations in relapse. Bar chart at the top panel illustrates the number of mutations found in each sample, highlighting the mutational gain in relapse.

Among the FP-RMS cohort (*n* = 18 patients), matched genomic data from at least two tumor samples was analyzed, including 10 MSK-IMPACT, 7 WES, 1 targeted Dragon/WES. In 16 patients the tumors had a *PAX3::FOXO1* fusion, while in 2 a *PAX7::FOXO1* fusion. Overall the genomic landscape was stable, with pathogenic or likely pathogenic alterations being identified in 51 cancer genes, with 9 (18%) of them showing alterations being recurrent across different samples (*MYCN, CDKN2A/2B, CDK4, GLI1, AKT1, IGF2, MED12, NCOR2*) (Fig. 1). Half of the patients acquired additional alterations at relapse compared to primary tumor (median, 1, range 0-5 alterations). FP-RMS had a median of 2 alterations in the primary and 3 at relapse (range 0–7). Two cases harbored only the *FOXO1* fusion at primary tumor and subsequently acquired up to 4 new alterations at relapse (Fig. 1). The most common recurrent alterations present at diagnosis, also designated as ‘secondary major alterations’, were mostly mutually exclusive: *CDKN2A/2B* alterations (5/18, 28%, 4 homozygous deletions, 1 non-sense mutation associated with loss of heterozygozity), *MYCN* alterations, (4/18, 22%, 2 amplifications, 2 missense mutations), and *CDK4* amplifications (3/18, 17%, coamplified with *GLI1* in 2 tumors and *MDM2* in 1 tumor at 12q13-15 locus). In total 10 patients with such secondary major alterations (other than the fusion) in the primary tumor acquired zero or very few additional alterations at relapse (Fig. 1, left subgroup). In contrast, 8 patients lacking major secondary genetic alterations in the primary tumors developed a higher number of additional alterations in the relapse/metastatic disease (Fig. 1, right subgroup). Two tumors from this latter group acquired either *CDK4* amplification or *MYCN* alterations at relapse. *AKT1* deletions was the only other recurrent event which was only detected at relapse. The two tumors harboring the *PAX7::FOXO1* fusion transcript were associated with *FOXO1* amplification (Fig. 1).

**Fig. 2:**
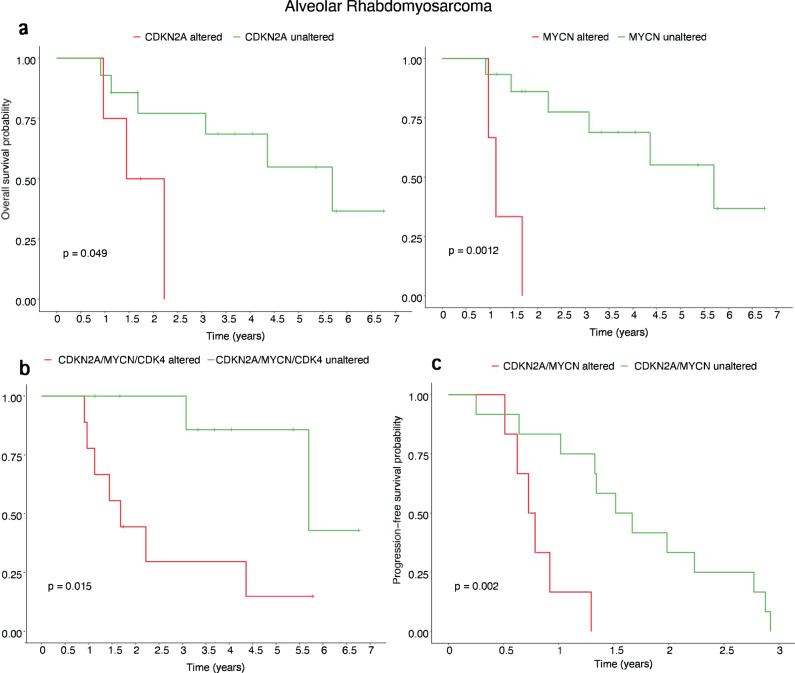
Overall survival (OS) and progression-free survival (PFS) genomic correlates in the FP-RMS cohort. Significant correlations in OS were found related to either **a** individual gene alterations in *CDKN2A* (*p* = 0.049) or *MYCN* (*p* = 0.0012), as well as **b** combined *CDKN2A* and *MYCN* alterations; **c** the presence of either of these two *CDKN2A or MYCN* alterations also correlated with PFS (*p* = 0.002).

We then tested the impact of several gene alterations on survival, including only alterations being recurrent accross different samples for the statistical analysis. Of interest, *MYCN* alterations (*p* = 0.0012) and *CDKN2A* deletions (*p* = 0.049) have a statistically significant impact on overall survival (OS) either alone or when combining both altered populations (*p* = 0.0017) (Fig. 2a, b). The presence of either *MYCN*, *CDKN2A,* or *CDK4* alterations were also associated with worse OS (*p* = 0.015). For progression-free survival (PFS), *CDKN2A* deletions (*p* = 0.0082) alone or in combination with *MYCN* altered tumors (*p* = 0.002) were found to have an unfavorable outcome (Fig. 2c). No differences in OS and PFS were observed when comparing the patients with major secondary genetic alterations at baseline versus those lacking secondary events in the primary tumors which subsequently acquired new alterations at relapse.

In the FN-RMS cohort (*n* = 17 patients) paired primary and relapse samples were tested by either targeted or whole exome sequencing, including 10 MSK-IMPACT, 6 WES, 1 targeted Dragon/WES. A total of 113 different genes were found to harbor either somatic pathogenic or likely pathogenic mutations (Supplementary Figure 1). Sixty alterations occurred at diagnosis and 133 alterations in the relapse (111% overall increase), with an average of 4.3 newly acquired alterations per patient (range 0–17) and 5.6 mutations per tumor sample (range 0–20). Two-thirds of the tumors (13/17) showed new genetic alterations in the relapsed tumors, with 4 tumors acquiring >10 new mutations (Supplementary Figure 1).

In contrast to FP-RMS, the genomic landscape in FN-RMS showed an even distribution between copy number changes (59%) and SNV (41%): 83 amplifications, 32 deletions, 61 nonsynonymous SNV, 6 non-sense SNV, 1 splicing-site variant, 3 frameshift insertions, and 10 frameshift deletions. Overall the average number of mutations per tumor sample was higher in FN-RMS vs FP-RMS (5.6 vs 3.2, *p* = 0.002).

**Fig. 3:**
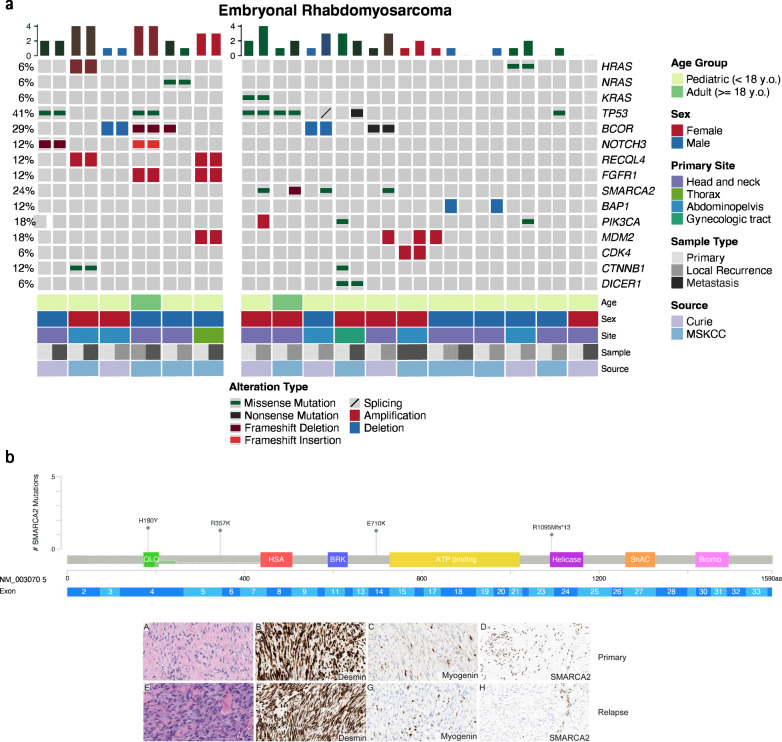
Oncoprint representation of SNV or CNV alterations in the entire FN-RMS cohort (35 samples, 17 patients). **a** Oncoprint representation of SNV or CNV in a subset of FN-RMS with detectable genetic alterations. Frequency of gene alterations (left column, %) is applied to the whole sample cohort per patient (*n* = 35 tumors, 17 patients). Samples from same patients are grouped together and color-coded (shades of gray) based on primary tumor, local recurrence, or distant metastasis. Left side plot groups the patients without newly acquired major alterations at relapse and right side plot depicts patients with clonal evolution. Bar chart at the top panel shows the number of alterations in each sample highlighting the mutational gain in the relapse. One patient lacking mutations in this oncoprint is further detailed in Supplementary Figure 1. **b** Lollipop plot of *SMARCA2* SNV mutations found in 4 relapsed FN-RMS, depicted in association to corresponding protein domains. Lower panel showing histology of primary/relapsed FN-RMS with *E710K* mutation (H&E, A, E), and immunohistochemistry positivity for desmin (B, F) and myogenin (C, G) and loss of SMARCA2 protein expression (D, H).

Among the recurrently altered somatic genes were *TP53* (7/17 patients, 41%), *BCOR* (5/17, 30%)*, PIK3CA* pathway genes (*HRAS, NRAS, KRAS, PIK3CA*) (5/17), *MDM2* (4/17), and *SMARCA2* (4/17) (Fig. 3a). *BAP1* deletions (2/17) and *SMARCA2* missense mutations and frameshift deletions (4/17) were found only during relapse. *SMARCA2* alterations were the most common events acquired in relapsed FN-RMS, with 4 different point mutations found (VAF < 50%) with no redundancy in protein domain. Immunohistochemistry performed in two tumors with available material showed complete loss of SMARCA2 protein expression (BRM antibody, Cell Signaling Technology, D9E8B clone, 1:1000) in one (Fig. 3b) and partial loss in a second. No other mutations of the SWI/SNF complex or associated copy number changes were found.

One patient showed no pathogenic or likely pathogenic somatic mutations by WES on primary tumor and acquired a single deletion of 30Kb in dystrophin (*DMD)*. Alterations in the DNA repair genes (*RAD51B, RAD21, RAD51C, ATR, RNF, ATM, FANCA, FANCC*) were exclusively present at relapse and associated with higher mutational counts (*n* = 3 cases) (Supplementary Figure 1).

We also analyzed the MSK FN-RMS cohort for potential recurrent whole chromosomal gains. Among the 10 FN-RMS investigated: 3 showed whole chr2 gain, 4 showed whole chr5 gain, 6 showed whole chr8 gain, 3 showed whole chr12 gain and 3 showed whole chr14 gain (Supplementary Figure 2). These changes appeared consistent in both primary and relapse samples.

We then tested the survival impact of several gene alterations on OS or PFS in the cohort with FN-RMS, however, none were identified. Additionally, no statistically significant difference was noted on survival in patients with FN-RMS having higher mutational count at relapse, by comparing cases with tumors acquiring >1 and >5 new mutations (*p* = 0.12 and *p* = 0.63, respectively). Furthermore, no differences in OS and PFS were observed comparing FN-RMS with or without acquired new alterations at relapse.

### Primary and metastatic/relapsed tumor samples—sequential RNA sequencing and expression data analysis

This analysis compared the expression levels of genes of interest (*MYCN, CDK4*, and *CDKN2A*) to their specific type of DNA alteration. Results showed that FP-RMS with *MYCN* amplifications were associated with high levels of *MYCN* mRNA expression, at comparable levels with other tumors harboring *MYCN* amplifications (Supplementary Figure 3).

### Primary and metastatic/relapsed tumor samples—mutational signature analysis

Next, de novo analysis of single base substitution (SBS) mutational signatures comparing primary and recurrent cases among FP-RMS and FN-RMS from the MSKCC cohort was performed. Thereafter, these mutational signatures were compared to reference COSMIC mutational signatures^8^ (https://cancer.sanger.ac.uk/signatures/sbs/). Defective DNA mismatch repair SBS signature was seen only in recurrent FP-RMS, but was seen in both primary and recurrent FN-RMS (Supplementary Table 2).

### Circulating tumor DNA analysis

A total of 62 plasma samples were collected from 10 patients based on availability, including 44 blood and 18 bone marrow aspirates (median of 7 samples/patient, range 3–10). The samples were linked to a median of 6 different timepoints per patient (range 2–9). For some timepoints both blood and bone marrow were available. Ten cases were included in the final analysis, 7 patients with FP-RMS (48 ctDNA samples, 45 timepoints) and 3 with FN-RMS (14 ctDNA samples, 12 timepoints), with 8 cases having timepoints spanning both diagnosis and relapse. The 2 remaining cases (1 FP-RMS, 1 FN-RMS) had ctDNA samples available only from relapse timepoints.

We then compared the variant detection sensitivity in the setting of confirmed active disease, showing a significantly higher rate in plasma derived from blood (86%) compared to the bone-marrow plasma (61%). There were no false positive ctDNA results detected in patients lacking active disease (*n* = 5).

Ten patients (7 FP-RMS, 3 FN-RMS) were selected as having alterations that could be tracked by liquid biopsy, including 7 fusions, 6 SNV, and 8 focal CNV (further detailed in Supplementary Table 3), and having ctDNA detected in at least one of their timepoints. DNA-based targeted custom panel detected an average of 2.8 variations per sample (median 2, range 1–11), which were then referenced to the baseline tumor genotype. In the primary tumor, the SNVs selected as informative had a wide range (7–65%) of allelic ratios (AR).

We first analyzed the variant detection rate at diagnosis in the 8/10 cases with available timepoints (Table 2, Supplementary Table 4). For the 6 FP-RMS, overall alterations were found in all plasma samples at diagnosis using our combined approach: the *PAX3::FOXO1* fusion (6/6), and SNV (2/2), while CNV were found in 80% (4/5) of patients. Among the 6 FP-RMS, 2 primary tumors harbored SNV at diagnosis (*RB1, PIK3CA*, and *CDKN2A* alterations) that could be screened in the plasma and were successfully detected in the ctDNA at diagnosis (Table 2). Similarly, 80% of CNV detected in the primary tumors were confirmed at ctDNA level, including *CDK4* gains/amplification, *MYCN* amplification, and *CDKN2A/2B* deletion. The overall detection rates for all timepoints per alteration type are described in Supplementary Table 5.

**Table 2:** ctDNA detection rates at diagnosis (*n* = 8).

Patients *N* = 8	Fusion detection	Fusion detection ratio	SNV detection	SNV detection ratio	CNV detection	CNV detection ratio	Combined approach detection ratio
	/	/	/	/	/	/	88% (7/8)
FP-RMS1	**Yes**	100% (6/6)	na	100% (2/2)	*CDKN2A* deletion	80% (4/5)	100% (6/6)
FP-RMS3	**Yes**		***RB1, PIK3CA***		na		
FP-RMS4	**Yes**		na		***CDK4*** **gain**		
FP-RMS5	**Yes**		na		***CDK4*** **Gain*; CDKN2A*** **deletion**		
FP-RMS6	**Yes**		na		***CDK4*** **amplification**		
FP-RMS7	**Yes**		***CDKN2A***		***MYCN*** **amplification**		
FN-RMS6	na	na	***BCOR, HRAS***	100% (2/2)	***MDM2*** **amplification**	100% (2/2)	100% (2/2)
FN-RMS8	na		na		***BCOR*** **deletion**		

Variants detected are in bold font; na, not available.

The two FN-RMS cases with available timepoints at diagnosis harbored variant alterations (*HRAS* hotspot nonsynonymous mutation and *BCOR* stopgain) and CNV (*MDM2* amplifications and *BCOR* deletion) that were successfully detected in ctDNA (Table 2).

A similar analysis was then performed in the relapse setting on the entire cohort of 10 cases (Table 3). For the 7 cases with FP-RMS, *PAX3::FOXO1* fusion was detected in 86% (6/7), SNV in 67% (2/3), and CNV in 67% (4/6). Specifically, *CDKN2A* deletions and *MDM2* or *MYCN* amplifications were detected in all cases. *CDK4* amplifications were detected in 2/4 cases harboring this alteration. In total, the ctDNA tool captured 86% (6/7) of FP-RMS variants at relapse. The three FN-RMS had an overall detection of at least one variant in 100% of samples. An *NRAS* nonsynonymous SNV was detected, while mutations in *BCOR* and *HRAS* were not. CNVs were detected in both cases including *MDM2* amplification or *BCOR* deletion.

**Table 3:** ctDNA detection rates at relapse.

Patients (*n* = 10)	Fusion detection	Fusion detection ratio	SNV detection	SNV detection ratio	CNV detection	CNV detection ratio	Combined approach detection ratio
							90% (9/10)
FP-RMS1	**Yes**	86% (6/7)	na	67% (2/3)	***CDKN2A*** **deletion**	67% (4/6)	86% (6/7)
FP-RMS3	**Yes**		***RB1, PIK3CA***		na		
FP-RMS4	**Yes**		na		*CDK4* gain		
FP-RMS5	**Yes**		na		***CDK4*** **gain*; CDKN2A*** **deletion**		
FP-RMS6	No		na		*CDK4* amplification		
FP-RMS7	**Yes**		***CDKN2A***		***MYCN*** **amplification**		
FP-RMS8	**Yes**		*MYCN*		***CDK4*** **amplification**		
FN-RMS3	na		***NRAS, FBXW7***	50% (1/2)	na	100% (2/2)	100% (3/3)
FN-RMS6	na		*BCOR, HRAS*		***MDM2*** **amplification**		
FN-RMS8	na		na		***BCOR*** **deletion**		

Variants detected are in bold font; na, not available.

We then analyzed the detection rates of each technique focusing only on ctDNA timepoints collected from clinically active disease, after reviewing patient charts, regardless if present at primary or relapse, or type of therapy. (Supplementary Table 5). For FP-RMS, we selected 40 ctDNA timepoints from the 7 patients in which the *PAX3::FOXO1* fusion detection was expected, 27 timepoints from the 5 patients with a selected SNV and 44 timepoints from the 8 patients with a gene-level CNVs (Supplementary Table 3). Detection rates were 83% (33/40) for *PAX3::FOXO1* fusion, 67% (18/27) for SNV, and 66% (29/44) for CNV.

**Fig. 4:**
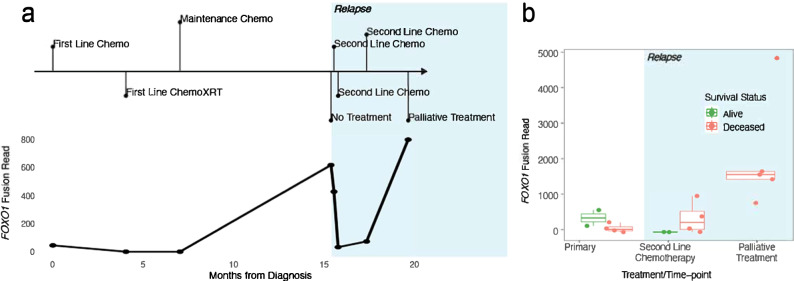
ctDNA evaluation in FP-RMS. **a** A FP-RMS clinical timeline with different timepoints throughout the treatment course showing correlation of ctDNA *PAX3::FOXO1* fusion reads number with tumor progression. **b** Box-plot depicting number of fusion reads related to final clinical outcome and timepoint of detection (red, deceased patients; green, alive). *FOXO1* fusion read numbers were higher in patients following a poor outcome (*n* = 7 patients). Chemo, Chemotherapy; XRT, radiotherapy. Center line corresponds to the median; lower and upper hinges correspond to 25th and 75th percentiles; upper and lower whiskers correspond to 1.5 x inter-quartile range.

**Fig. 5:**
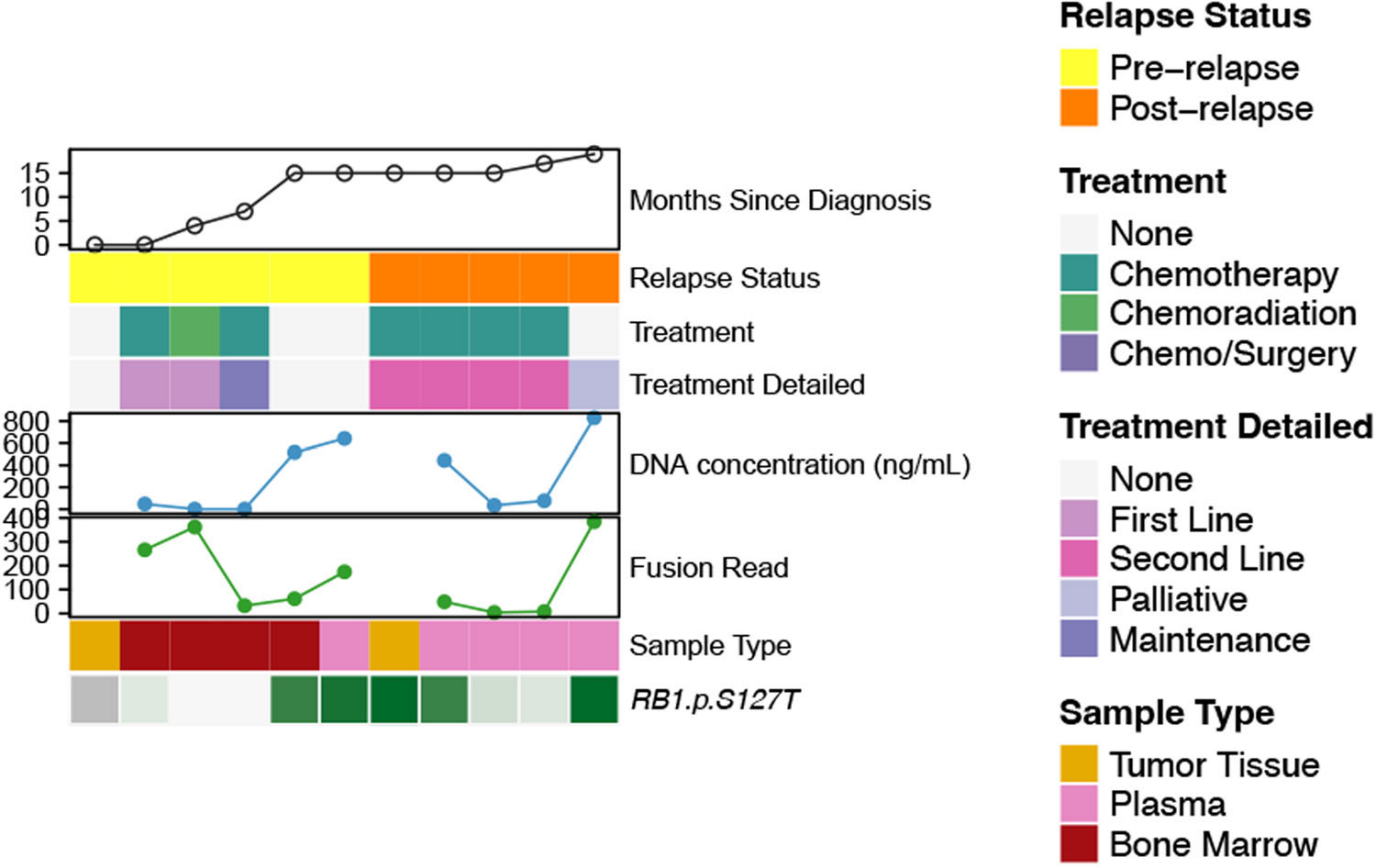
Composite representations of ctDNA findings in a FP-RMS patients for fusion reads and SNV detection. Clinical timelines, relapse status, and treatment course are color-coded. Liquid biopsy details include cfDNA concentration (blue curve), sample type, fusion read number and other genomic data. Upper panel (green curve): fusion read number decreases during first- or second-line therapy, then gradually increases as the patient develops treatment resistance, with major read number escalation at palliative care stage. Lower panel (shades of green heatmap): showing *RB1* SNVs detected at relapse and identified throughout patient-care, following a similar detection pattern variation as observed with the fusion reads number. ctDNA SNVs detected by DNA-based custom targeted panel (4000X coverage); color-coding (green) intensity relates to the variant allele ratio (VAR) in plasma. Data not available for primary tumor (first column) in this patient.

**Fig. 6:**
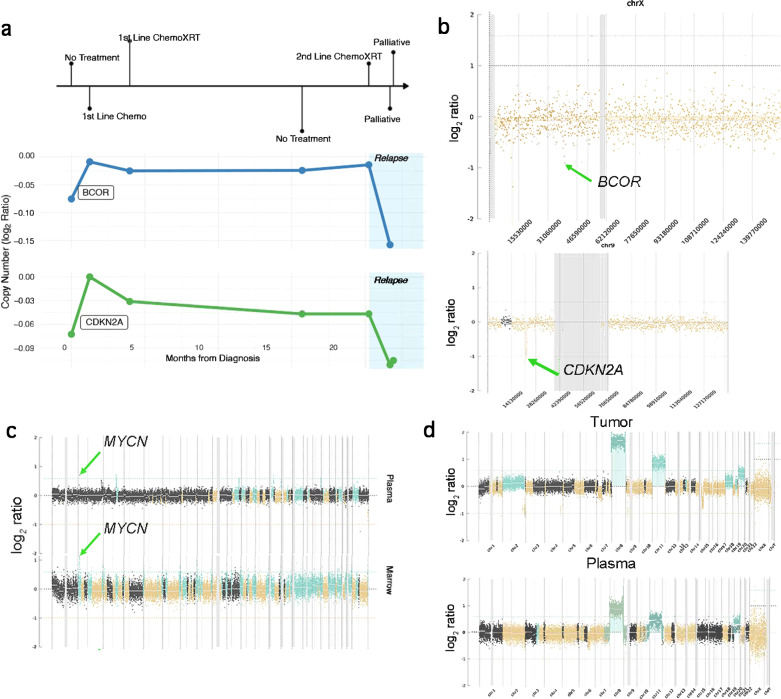
ctDNA CNV detection in FP- and FN-RMS. **a** Clinical timeline with different timepoints throughout treatment course in a FN-RMS showing *BCOR* and *CDKN2A* copy number changes (log_2_ ratio) in plasma using shallow (4X) WGS correlated with disease burden. Baseline WES from the same patient tumor showed 2Kb Xp11 deletions and 650Kb 9p21 involving *BCOR* and *CDKN2A* loci, respectively. **b** Same patient whole chromosome X and whole chromosome 9 visualization confirming the ctDNA CNV changes in *BCOR* and *CDKN2A* loci. **c** Comparative whole-genome visualization of copy number changes (log_2_ ratio) by shallow WGS in a FP-RMS using two different sample types at the time of diagnosis (blood and bone marrow) confirming the *MYCN* gene-level amplification (green arrow) detected in the tumor profile at baseline (chr2p24.3 1.4 Mb *MYCN* amplification). **d** Comparative whole-genome visualization of copy number changes (log_2_ ratio) derived from shallow WGS from the same FN-RMS illustrated in (**a**), (**b**), on matched tumor sample and blood at diagnosis showing a similar arm-level CNV profile. WES of primary tumor showed whole chromosomal copy number gain (green) of chromosomes 8, 11, and 20. Chemo, chemotherapy; XRT, radiotherapy.

Finally, we aimed to assess if fluctuations of ctDNA variant detection is linked to disease status and can be used to monitor treatment response or disease progression. We tagged each timepoint to a specific clinical context: diagnosis, during first-line treatment (chemotherapy, radiotherapy, and/or surgery), follow-up, relapse, during second-line treatment, and palliative care. First, there was no correlation noted between cfDNA concentration variations and clinical context. Second, we used unique molecular identifiers (UMI) for fusion identification during deep panel sequencing, which allowed a quantitative comparison of ctDNA levels from various timepoints based on the read number. The results showed that the number of fusion reads was closely linked to the clinical context in all 7 FP-RMS. One of them is depicted in Fig. 4a. All cases had increased fusion reads number at higher disease burden timepoints as well as at relapse in patients following a fatal outcome (Fig. 4b). In 2 patients with sufficient timepoints available, VAF fluctuations of SNVs showed a trend towards higher ratios with increased disease burden and lower ratios during follow-up periods (Fig. 5). Similarly to both previous observations, CNV fluctuation levels recapitulated the clinical context (Fig. 6a). These alterations were detected by shallow WGS CNV profiles (Fig. 6b), with plasma from either blood or bone marrow providing satisfactory cfDNA CNV detection (Fig. 6c). Moreover, the cfDNA whole genome view recapitulated the CNV profile identified in the primary tumor (Fig. 6d).

## Discussion

The overall survival for patients with recurrent RMS is extremely poor, with a heterogeneity of outcomes being observed^9^. Moreover, our current understanding on how tumors evolve in the context of standard of care therapy remains limited. Also undetermined is the potential causal link between somatic mutations at baseline diagnosis, including tumor mutational burden (TMB), and the risk for treatment resistance and relapse. These shortcomings come from the paucity of comprehensive genomic studies investigating sequential tumor samples along the oncologic progression. In this study, we undertake a longitudinal genomic analysis to comparatively investigate the clonal evolution upon metastatic disease or relapse in a group of FP-RMS and FN-RMS. The study follows a two-level approach. First, we seek to characterize the acquired mutation patterns upon drug failure and metastasis by targeted DNA sequencing or WES in order to define genomic outlines of relapse that can be monitored through liquid biopsy. Second, we use a multisample and multiplatform NGS strategy in liquid biopsies to define feasibility and best methodology approach for disease monitoring during clinical progression.

Prior genomic investigations in FP-RMS have shown that a subset of tumors harbor so-called ‘secondary genetic events’ that may cooperate with the primary *FOXO1* fusion driver, resulting in different clinical impact^7^. Thus, a number of studies showed the presence of recurrent alterations, such as *MYCN* and *CDK4* amplifications^4–7^ in the 10–15% incidence range, however, their role in risk stratification remains controversial. *MYCN* overexpression and copy number gain was initially reported to be associated with unfavorable outcome in FP-RMS^10^, however, subsequent studies have not confirmed this finding either by FISH or NGS^6,7^. Similar discrepant findings relate to *CDK4* amplifications which were initially found to be predictive of outcome^7^, but refuted more recently in a larger study of 151 FP-RMS by NGS^6^. In the current cohort, we show that the most common secondary genetic alterations in primary FP-RMS are *CDKN2A* deletions (28%), *MYCN* alterations (28%), and *CDK4* amplifications (22%), mostly present at baseline diagnosis and found to have an impact on survival (both OS and PFS). It is important to highlight that the incidence of these alterations in our study is significantly higher than in other investigations, likely related to the nature of our cohort, restricted to patients who developed recurrence. Additional prospective large-scale studies are needed to confirm the prognostic value of these events in high risk, relapsed FP-RMS. Moreover, the impact of these secondary events in triggering relapse or resistance to therapy has not been investigated to date. Interestingly, in our cohort, tumors harboring these major secondary genetic alterations at diagnosis harbor no or very few acquired alterations at relapse, suggesting a potential mechanism in driving progression. This contrasts with FP-RMS lacking defined secondary hits that subsequently developed new genetic alterations in the recurrence. These observations remain intriguing and require further validation in a larger number of cases.

The genomic landscape of FN-RMS is more heterogeneous, with a number of potential genetic drivers being implicated in their pathogenesis, including alterations in tumor suppressor genes (*BCOR, NF1*, and *TP53*), *RAS* activating mutations or *FGFR1* amplifications^5,6^. Here we report that the most common events are loss of function alterations in *TP53* (33% samples, 41% patients) and *BCOR* (24% samples, 30% patients). These alterations were reported at significantly lower rates in other series (7–15% range), likely due to our study design focusing on patients with relapsed FN-RMS^6,11^. Although some studies^4,12^ found *CDKN2A* homozygous deletions in 20–25% of FN-RMS, neither Shern et al.^6^ nor our results confirmed this finding. Among these alterations, only *TP53* mutations have been associated with poor outcome^6^, however, no recurrent molecular alterations in our FN-RMS cohort correlated with survival. Similar to the FP-RMS subset, we show that FN-RMS acquire no or very few mutations at relapse if the primary lesions harbored one of the defined driver events (*BCOR, NF1, FGFR1, RAS*), except in the *TP53* mutations setting. CN-profiles in FN-RMS show recurrent whole chromosomal gains (i.e., chr 3, 5, 8, 12, and 14) present from baseline, which recapitulate prior findings in the literature using low resolution cytogenetic methods^13,14^. Moreover, our mutational signature analysis comparing primary (pre-treatment) and recurrent (post-treatment) FP-RMS and FN-RMS cases show a defective DNA mismatch repair single base substitution signature only in recurrent FN-RMS, while being detected in both subsets of FP-RMS.

These results unveil certain mutation patterns present only at relapse in FN-RMS, including *BAP1* deletions (2/17, 12%) and *SMARCA2* missense mutations and frameshift deletions (4/17, 24% patients). SMARCA2 is a member of the SWI/SNF chromatin modulating complex, and together with SMARCA4 are mutually exclusive subunits responsible of its ATPase activity^15,16^. Alterations in the SWI/SNF complex represent one of the most frequent somatic mutations in cancer^17,18^. While further investigation is needed, these results suggest that alterations in the SMARCA2 unit may play a key role in FN-RMS relapse evolution.

A subset of RMS is driven by coexisting alterations in multiple genes suggesting an evolutionary selection of multiple subclones that may drive relapse or refractory disease^19^. Only one study to date has specifically investigated intratumoral heterogeneity among both FP-RMS and FN-RMS using whole-genome sequencing (WGS). The results showed significant heterogeneity in 10 of the 15 cases tested, with few mutations being detected in the ‘founding clone’, while additional subclones carried the majority of SNV detected^19^. Moreover, the same study tested clonal evolution following treatment in two FN-RMS patients, investigating the mutational allele frequency (MAF) to distinguish de novo SNVs in the recurrent samples from mutations at low frequency in the primary tumor^19^. Their results showed that in both patients, the major clone from the primary tumor was eliminated following initial line of therapy, while the minor clone from the primary acquired additional SNVs and evolved into two major clones at recurrence. This suggests that chemotherapy provides a selection pressure that favors enrichment of treatment-resistant clones over time. Our longitudinal sampling of diagnostic and recurrent tumors with ctDNA along the treatment course also detects the emergence of treatment-resistant clones harboring mutations with high VAF not detected in the earlier timepoints, but apparent in the ctDNA and tumor relapse following several courses of chemotherapy (Fig. 5). Another study investigated clonal deconvolution and phylogenetic analyses using WGS and WES on multiregional tumor sampling in three common pediatric tumors, including RMS^20^. Their findings showed significant intratumoral genetic heterogeneity and a direct correlation between the number of clonal branching with risk of malignancy.

Liquid biopsies have recently gained popularity as minimally invasive, easy to perform, and repeatable approaches. Highly sensitive and specific methods to detect ctDNA are emerging in various cancer types for either single-nucleotide variations or whole-genome sequencing to establish copy-number changes^21^. However, these investigations have mainly been performed in epithelial malignancy and remain exceedingly rare in RMS. Two recent publications focused on the detection feasibility in RMS^22^ and risk stratification in intermediate-risk RMS^23^, while another mainly in isolating circulating tumor cells^24^. An additional study focusing on recurrent/refractory malignancies in pediatric and young adult patients, including RMS, found a 57% detection rate of SNV in circulating cell free DNA (cfDNA) and matched tumor by WES, including a 76% rate of actionable alterations^25^. However, this study was limited to SNV detection and did not assess CNA or structural rearrangements, while other studies have specifically developed bioinformatic tools to detect them more accurately in pediatric sarcomas with low mutational burden^26^.

The current work is designed as a pilot study to evaluate the best genomic strategy that can be applied in clinical settings for using liquid biopsy in treatment monitoring. A comprehensive methodology of both translocation breakpoints and SNV detection by deep targeted sequencing, and CNV assessment by shallow whole-genome sequencing is employed to establish the feasibility and sensitivity of each platform applied on retrospectively available plasma or bone marrow aspirates. Overall, the results show a higher sensitivity using plasma derived from blood rather than bone marrow aspirates, with true-positive detection rates of 86% and 53%, respectively. We first confirm the high sensitivity of our combined approach in both FP and FN-RMS subtypes at diagnosis, being able to detect the *PAX3::FOXO1* fusion and SNVs in 100%, and CNVs in 86% of the cfDNA timepoints. Our tool efficiently detects gene-level copy number gains/amplifications in *CDK4, MYCN*, and *MDM2*, or losses in *CDKN2A* and *BCOR*. Similarly, in the relapse setting, 86% of FP-RMS and 100% FN-RMS were correctly confirmed to harbor such alterations. This data provides solid rationale to implement liquid biopsy in both RMS subtypes, using the two screening platforms, including the high coverage custom targeted DNA sequencing for detecting gene fusions and SNV, shallow WGS for CNV alterations.

Previous studies have described an apparent correlation between cfDNA concentration and clinical status and tumor size^27,28^, however, this was not evident in our results. Similarly, Andersson suggested that cfDNA levels may be misleading and could result in over- or under-estimation of tumor burden due to normal fluctuations and advised relying on somatic mutation detection^29^. Moreover, the ctDNA content in cfDNA appears to vary significantly between cancer types, with highest values being obtained in neuroblastoma compared to all sarcoma samples^25^.

Although fusion detection in FP-RMS has been previously investigated^27,28^, this collection is the largest longitudinal ctDNA sequencing effort to date. A somewhat similar approach in cfDNA was adopted by Klega et al. using a hybrid capture bait for a translocation-specific sarcoma sequencing (TranSS-Seq) assay, designed to detect oncogenic gene fusions as well as mutations in common tumor suppressor genes (*TP53, STAG2*)^27^. Their TranSS-Seq detected ctDNA in 5 of the 7 FP-RMS tested, which were confirmed to harbor a *PAX3::FOXO1* fusion. Similarly, the study by Shah et al. using a CAPP-Seq targeted gene panel approach showed high sensitivity of detecting translocations in ctDNA of 3 FP-RMS samples^28^. Although digital droplet PCR and other tools may be more effective in detecting fusions, particularly in cases of low disease burden, our integrated genomic platform has the versatility to detect the presence of fusions, SNVs, and CNVs, offering a reliable conduit to overcome tumor heterogeneity. However, it may be more suitable for high-risk or high-disease burden setups, as previous studies suggested a correlation between lower risk and lack of ctDNA detection^30^, which could not be interrogated in this analysis.

Notably, we demonstrate a correlation between fusion reads and clinical context, with a higher number of reads being detected in the presence of increased disease burden, while a lower read number was found in all FP-RMS showing treatment response (*n* = 7). One limitation of our study design is the inability to specifically address if ctDNA can detect relapse before it is detected clinically or radiographically. However, in one patient the fusion read detection was concomitant with the diagnostic imaging of relapse, suggesting a potential role in monitoring clinical progression. This is particularly relevant as recent data suggest the need for innovative disease follow-up methods, as routine imaging has not demonstrated improved outcomes^31^. Moreover, these findings show correlations between the clinical context and SNV VAFs or CNV log2 ratios of ctDNA variant alterations. Prior direct correlations between clinical course and variant ctDNA levels have only been anecdotally assessed in RMS^28,32^. In contrast, this paradigm has been better established in other pediatric cancers such as Ewing sarcoma^33,34^, and Wilms tumors^35^. While these findings are highly encouraging, one major bias is the retrospective nature of our sample collection and lack of a centralized processing of peripheral blood samples. The latter confounding factor led to exclusion of 4 cases (1 FP-RMS, 3 FN-RMS) for which no ctDNA could be detected in the 11 samples combined. A recent study revealed an association between ctDNA levels and unfavorable location and metastasis in RMS^22^. A subsequent study focusing on intermediate-risk RMS demonstrated a correlation between clinical outcome and ctDNA detection at diagnosis, utilizing a tool similar to ours^23^.

In summary, these findings support the benefit of future implementation of liquid biopsies for screening real-time disease burden and treatment response in both RMS subtypes. We show for the first time through a combined platform approach that ctDNA is a reliable tool in detecting *PAX3::FOXO1* fusion, SNVs and gene level copy number variation in both FP-RMS and FN-RMS. Our pilot investigation provides a rationale for implementing ctDNA analysis in a prospective international ancillary study for children and adults with frontline and relapsed RMS (FaR-RMS, NCT04625907)^36^. One goal relies in a prospective and systematic collection of peripheral blood samples for cfDNA extraction at each patient-care milestone to confirm its role in treatment response monitoring, therapeutic target identification, and relapse prediction. Furthermore, patients with RMS tumors showing alterations in *CDKN2A, CDK4*, and *MYCN* for FP-RMS and *TP53, BCOR*, or *RAS* pathway for FN-RMS may benefit from a shorter time-lapse between cfDNA collections during follow-up. This study also demonstrates the feasibility and added value of trans-Atlantic collaborations, especially in pediatric sarcoma, often limited by their rare incidence for meaningful research. Moreover, the multiplatform approach highlights the reproducibility of these results.

## Methods

### Patient Selection

The files of the Department of Pathology at Memorial Sloan Kettering Cancer Center (MSKCC) (2013–2022) and the Pediatric Department at SIREDO/Institut Curie (IC) (2003–2020) were searched for patients with metastatic/relapsed RMS in which next generation sequencing (NGS) was performed on multiple (at least two) tumor samples. The study was approved by the Institutional Review Board (IRB) and ethics committees at both institutions and all participants provided written informed consent to take part in the study (MSKCC IRB# 02-060, Institut Curie DATA210014). We included patients having a confirmed pathologic and molecular diagnosis of metastatic/relapsed RMS for whom sequential NGS data was available, in most cases including primary and relapse samples. Ten cases underwent molecular analysis as part of the European MAPPYACTS trial^25^. All FP-RMS samples were confirmed for the presence of *FOXO1* fusion either by RNAseq (IC) or Archer FusionPlex (MSKCC)^37^. A total of 35 patients were selected from both institutions (20 from MSKCC, 15 from IC), which included both pediatric and young adults. Pathology reports and clinical charts were reviewed for the tumor size, treatment information, date and location of metastatic disease or relapse, last follow-up, and status of disease. As Children Oncology Group (COG) and European Pediatric Soft Tissue Sarcoma Study Group (EpSSG) groups do not abide by the same risk stratification criteria, patients from the European sub-group were re-classified following the COG criteria allowing for a uniform data analysis. Approved treatment guidelines for this cohort were EpSSG-RMS2005 or MMT-95, D9602, D9802, D9803, ARST0331, ARST0431, ARST0531. To validate that the two patient cohorts have comparable outcomes, a statistical analysis was performed to estimate the median and its corresponding 95% CI for time-to-event data. Kaplan–Meier curves were compared using the log-rank test. Cox proportional hazards regression was used to identify prognostic factors for overall survival and event-free survival.

### Tumor and plasma samples

Collected tumor samples were formalin-fixed paraffin-embedded for MSKCC patients and frozen tissues for IC patients. RMS diagnostic was validated for each tumor sample by pathologic assessment and tumor cellularity was estimated as >20% for further analysis. Tumor cellularity was also inferred by bioinformatic analysis using Sequenza during WES^38^. Nucleic acids were extracted and quantified, and all samples were subject to QC before any further analysis with minimal requirement. Whole exome sequencing required 10-200 ng of DNA, while RNAseq required >1 µg RNA of sufficient quality (integrity number (RIN) > 4 and DV200 > 50%). Peripheral blood was used to extract normal germline DNA for every patient.

Due to the retrospective nature of our study, there was an inconsistent pattern of blood or bone-marrow samples collection. 16 patients with available frozen EDTA tubes from at least one relevant timepoint were selected. Samples were centrifuged at 1000 × *g* for 10 min to obtain 0.5–2.0 mL plasma aliquots which were then frozen at −80 °C. Samples were obtained from different/key timepoints throughout their clinical care, allowing to dynamically assess the feasibility of monitoring somatic alterations such as gene fusions, SNV, or CNV in the plasma. The cfDNA was extracted by Automated QIAsymphony kit for circulating DNA (Qiagen^®^ N.V., Venlo, Netherlands). The double-strand DNA (dsDNA) amount extracted varied depending on sample type. For blood, concentrations ranged from 2.7–383.7 ng/mL (median 47.4 ng/mL) while bone marrow samples concentrations ranged from 32.3–652.3 ng/mL (median 129.0 ng/mL). Samples were then assessed by nucleotide migration using cfDNA Screen Tape for 4200 TapeStation system (Agilent^®^) to provide a proportion between genomic (high molecular weight) DNA and cfDNA (expected 146 bp +/−20 bp). Size profiling revealed satisfactory cfDNA ratios for blood samples and expected high rates of contamination by genomic dsDNA (>1 kb) for bone marrow samples and overall lower ratios of cfDNA due to the usage of previously uncentrifuged frozen EDTA tubes. Around 5–50 ng of circulating cfDNA were processed with the pre-capture kit XT-HS2 (Agilent^®^) according to the manufacturers’ protocol. As ctDNA is estimated around 160–170 bp, no DNA fragmentation step was needed and size selection was done for most samples with gDNA contamination. This method was used both for plasma from blood and bone marrow aspirates. Libraries for all samples were then processed and barcoded similarly, quantified, and then qualified with the D1000 DNA ScreenTape analysis kit according to manufacturer protocols (Agilent®).

### Tumor samples molecular analyses

Detailed descriptions of MSK-IMPACT workflow and data analysis, a hybridization capture-based targeted NGS assay using matched tumor and blood-derived normal DNA were described previously^39^. This panel targets all exons and selected intron regions of 505 genes of interest in solid tumors. All mutational and copy number calls were generated by the standard MSK-IMPACT pipeline. Copy number amplification and deletion are defined as gains and losses of gene-level copy number greater than two-fold in the tumor relative to pooled FFPE normal based on NGS. Moreover, in order to investigate for recurrent whole chromosomal gains among FN-RMS, we analyzed CN profiles derived from MSK-IMPACT copy number of tiling probes comparing tumor to FFPE normal controls.

In addition, we performed mutational signature analysis using the VCF files for the primary (pre-treatment) and recurrent (post-treatment) MSK ARMS and ERMS cases in order to identify potential signature shifts or selection under chemotherapy. Mutational signatures were extracted using non-negative matrix factorization with analysis performed using the R package “sigminer” version 2.1.9 using VCF files as input^40^. De novo mutational signature discovery for single base substitution (SBS) was performed using the sig_unify_extract function with default parameters. Mutational signatures were then matched to SBSv3 reference mutational signatures in the Catalog of Somatic Mutations in Cancer (COSMIC) database using the get_sig_similarity function^8^.

Whole Exome Sequencing (WES) and Dragon targeted DNA-based panel sequencing. WES analyses at IC were performed on matched tumor samples and blood-derived germline DNA. 150–200 bp DNA fragments library was analyzed with SureSelect^XT HS^ kit and 67.3 Mb CRE v2 probes (Agilent®, Santa Clara, CA). DNA fragments were barcoded, captured, and enriched by PCR before quantifying and qualifying using 4200 TapeStation system. Fragments were then sequenced by NextSeq500 paired-end 2 × 100 bp (Illumina Inc., San Diego, CA). Mean sequencing depths of 200x for somatic and 100x for germline were reached.

Bioinformatic analysis was performed by alignment to the hg19 normal genome using Bowtie2 software. Single nucleotide variants (SNV) were detected using Genome Analysis Tool Kit (GATK) (HaplotypeCaller & UnifiedGenotyper) and MuTect2 calling from a 2766 curated cancer gene list. Exonic and splicing variants were analyzed and flagged by their COSMIC id, as well as filtering synonymous and germline variants. Variants were annotated using Annovar. SNVs were analyzed using VarSome 10.1^41^ and Alamut Visual Plus V1.4^42^. Although not standard in our bioinformatics analysis, SNV occurring at a low variant allele frequency (VAF < 5%) were filtered out when comparing key alterations between both cohorts for visualization purposes (unless present in a known oncogenic hotspot). A shortlist of SNV of either pathogenic or likely pathogenic was selected. Copy number variations (CNV) and loss of heterozygosity (LOH) were assessed by aligning the reads from tumor tissue to matched normal sample. Bioinformatic tools used were Sequenza (v2.1.0), DNAcopy (v1.52.0), and FREEC (v11.5) allowing data cross-checking.

In two cases from IC, Dragon DNA-based custom panel sequencing (571 cancer gene panel specific to soft tissue sarcomas and overlapping with the WES genes) (Illumina TruSeq Custom Amplicon) was performed instead of WES. The method has been previously described^43^. 50 ng of DNA input extracted from frozen tumors was used for library preparation with the same Agilent SureSelect XT-HS kit used for WES, according to the manufacturer protocol. This kit incorporates molecular barcodes (UMI), requiring high sequencing depths and enabling to reliably detect variants at very low allelic ratios. Samples were sequenced per 2 × 100 bp flowcell of the NovaSeq6000 Sequencer (Illumina^®^) to reach an average depth of 1500X and a minimum depth of 100X. Bioinformatics pipeline included quality control metrics determination and a variant calling using Varscan2 (v2.4.3).

RNA-Sequencing used a library construction from TruSeq Stranded mRNA Library Prep (Illumina Inc., San Diego, CA, Catalog#20020595) at IC. mRNAs were captured using poly-A beads, fragmented, and reverse transcribed into complementary DNA. Amplified libraries were then sequenced on NextSeq500 paired-end 2 × 150 bp (Illumina Inc., San Diego, CA).

Fusion detection was done from FASTQ files aligned on hg19 normal genome using two approaches: (A) targeted analysis, using an in-house tool designed to search for well-characterized fusion sequences; and (B) exploratory analysis attempting to capture fusion transcripts, using 5 fusion different detection tools: Defuse V0.6.2, StarFusion v1.2.0 (STAR v 2.5.4a), Fusion Catcher v1.00, FusionMap (Oshell toolkit v10.0.1.50) and ARRIBA v1.2.0.

Variant calling was performed by read alignment with STAR (v2.5.3a, on hg19 reference genome). Read cleaning was done as described by GATK good practices (v3.5) for marking duplicates, base recalibrations, and indel realignments. Variant calling was performed on a list of 499 genes (Cancer Gene Census, COSMIC 24.05.2016) using Haplotype Caller (GATK v.3.5) and Mutect2 (GATK v.4). Reads with mapping quality lower than 6 and sequenced bases quality lower than 20 were not considered for the variant calling. Variants were annotated with ANNOVAR (v2018Apr16) and formatted with the Python package hgvs (v1.2.5). Intra- and inter-analysis occurrence of variants were used to eliminate noise from the sequencing technology.

Expression data was aligned using Salmon and hg19 reference genome summarizing transcript expression levels as a table count of transcripts per million (TPM) using tximport (R library). The data were then used for hierarchical clustering analysis (Ward method and Spearman or Pearson correlation with or without interquartile range) and for boxplot generation. In parallel, the classification of samples to cancer subtypes was inferred using a variational autoencoder (VAE) model. This model includes expression data (generated with STAR v2.7.0e) of 21.916 cases already categorized (normal and cancer tissues) serving as control groups^44^.

RNA sequencing was available from 15 IC patients (8 FP-RMS, 7 FN-RMS). Several control groups available at IC were included for comparison, including a control FP-RMS group (*n* = 18), control FN-RMS group (*n* = 22), and other sarcoma types (Ewing sarcoma, 18, desmoplastic small round cell tumor, 12, synovial sarcoma, 18).

### Liquid biopsy genomic studies

The experimental design applied a comprehensive approach of both deep targeted and genome-wide shallow sequencing of several biologic samples. This strategy was employed using retrospectively available samples (plasma, bone marrow) from a high number of timepoints across the patients clinical course in order to evaluate the feasibility of using liquid biopsy to monitor therapeutic response.

RMS Targeted Custom Panel “CHEWIE” for SNV detection at high coverage for ctDNA.

A custom panel of 36 cancer genes was designed covering the most frequent mutations found in pediatric RMS, using published genomic studies (Supplementary Table 1). The design covered all exonic regions of known tumor suppressor genes, oncogenic hotspots, and described breakpoints involved in *PAX3::FOXO1* fusion. Coordinates were aligned on hg19 genome. The custom RMS panel was created spanning 26.520 120 bp-probes for a total size of 61.39 kb. Using unique molecular identifiers during circulating DNA sequencing process allowed to associate each read to a unique original molecule of cfDNA. Sequencing was done on NovaSeq6000 SP paired-end 2 × 100. Our design required a minimal sequencing depth of 49,000X for SNV detection in ctDNA.

The BAM files were obtained by alignment on the hg19 genome version and processed through our in-house pipeline. The data analysis was approached similarly to WES with prior filtering-out of overly recurrent variants, allelic ratios <5% (unless on a described oncogenic hotspot or present in the primary tumor), and synonymous SNVs. In the eventuality that the same c. mutation was detected in tumor and liquid biopsy, no minimal allelic ratio was required.

Shallow whole-genome sequencing (WGS) for ctDNA CNV Detection.

Following library preparation (day1 of the XT-HS2 kit, Agilent®), yet prior to hybridization and capturing for the CHEWIE panel, a small portion of extracted cfDNA was used for shallow WGS. 2 × 100 bp paired-end shallow sequencing was performed using an Illumina Novaseq6000. Raw reads were mapped by BWA MEM (v0.7.17) onto hg19 human reference genome. Reads overlapping with the RMS custom panel described above were removed using bedtools (v2.30.0) to avoid bias during normalization, as the shallow whole-genome sequencing was performed simultaneously to the targeted panel deep sequencing, using the same barcodes. The resulting BAM files were indexed by SAMtools (v1.14). A first analysis was performed with IchorCNA (v0.2.0), which includes a 1 Mb size of detection window and subsequently followed by WisecondorX (v1.2.4), to create normalized bins (100 kb), log2 ratios, for smaller pre-selected alterations. The reference used for this analysis was a subset of 25 germline samples. Predicted copy number alterations were annotated with a list of genes (*n* = 2767) frequently involved in cancer using bedtools.

### Reporting summary

Further information on research design is available in the Nature Research Reporting Summary linked to this article.

### Supplementary information

Supplementary Material and Results

Reporting Summary

## Data Availability

The anonymized MSKCC variant level data (mutation, copy number, structural variants including fusion) are available at: https://cbioportal.mskcc.org/study/summary?id=soft_tissue_msk_2023. The anonymized IC data is available at: https://ega-archive.org/ (EGA ID: EGAS00001007399). The data from the MAPPYACTS trial is available as specified by Berlanga et al.^25^. Any other relevant data is available from the authors upon request.
