# Supplementary material for: Sequential genomic analysis using a multisample/multiplatform approach to better define rhabdomyosarcoma progression and relapse
Source: NPJ Precis Oncol. 2023 Sep 20;7:96. doi: 10.1038/s41698-023-00445-1 (PMC10511463; doi:10.1038/s41698-023-00445-1)
Supplement: Supplementary file 1 — Supplementary Material and Results [file 41698_2023_445_MOESM1_ESM.pdf]

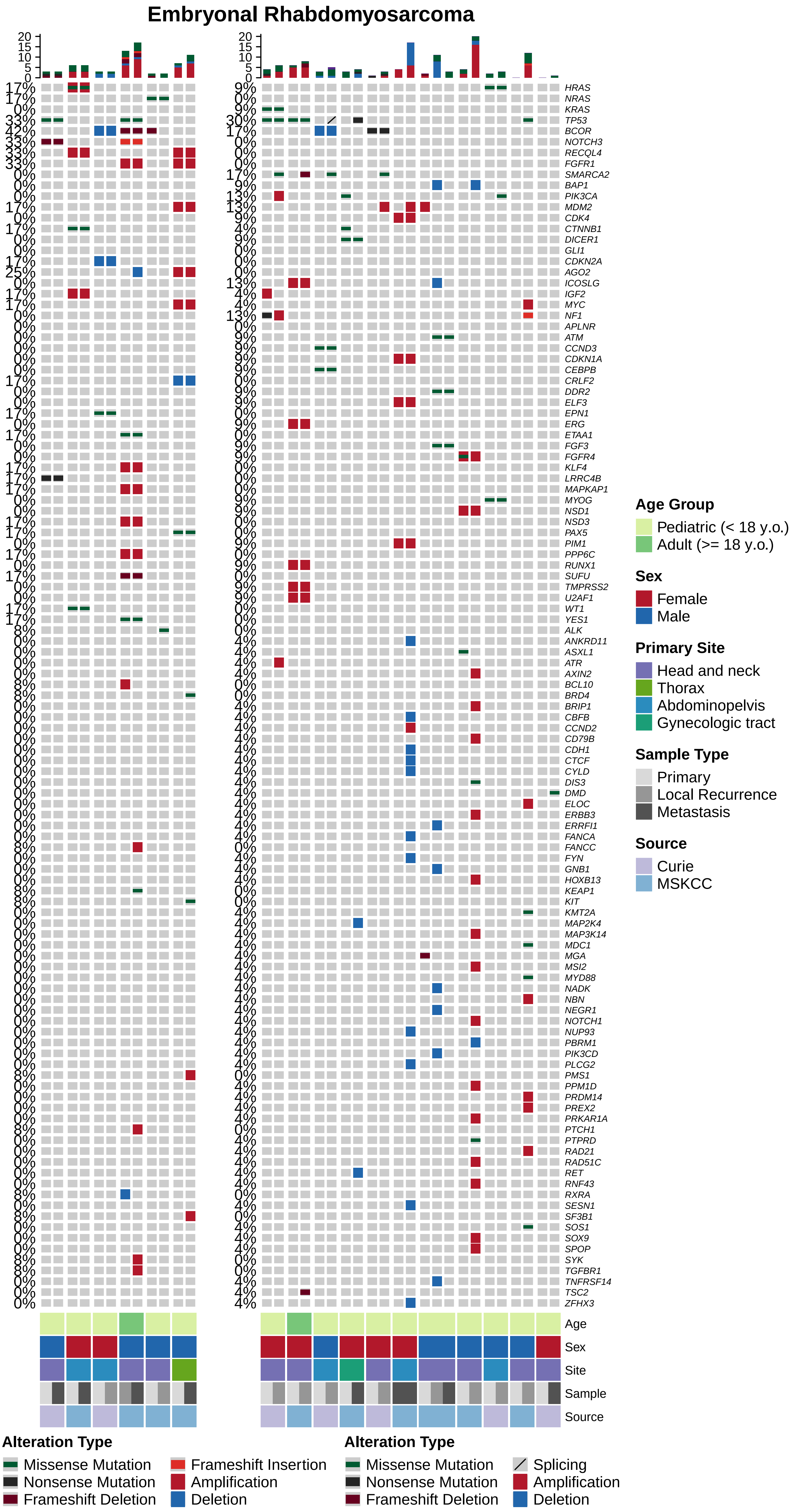

Supplementary Figure 1: Oncoprint of detected gene alterations in the entire FN-RMS cohort (33 samples, 17 patients).

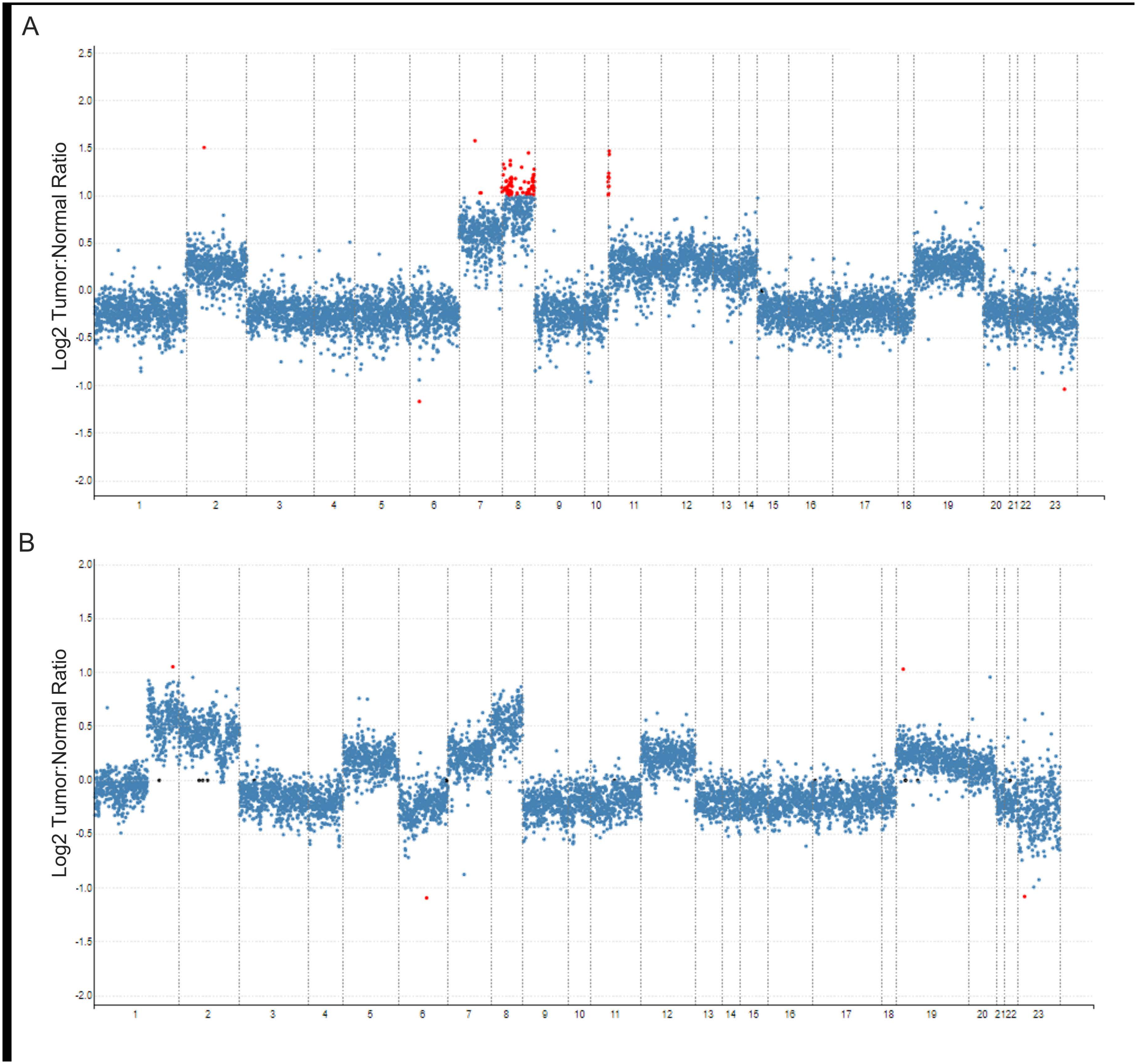

Supplementary Figure 2: Two examples of copy number profiles of FN-RMS showing whole chromosomal gains of chr2, 8, and 12.

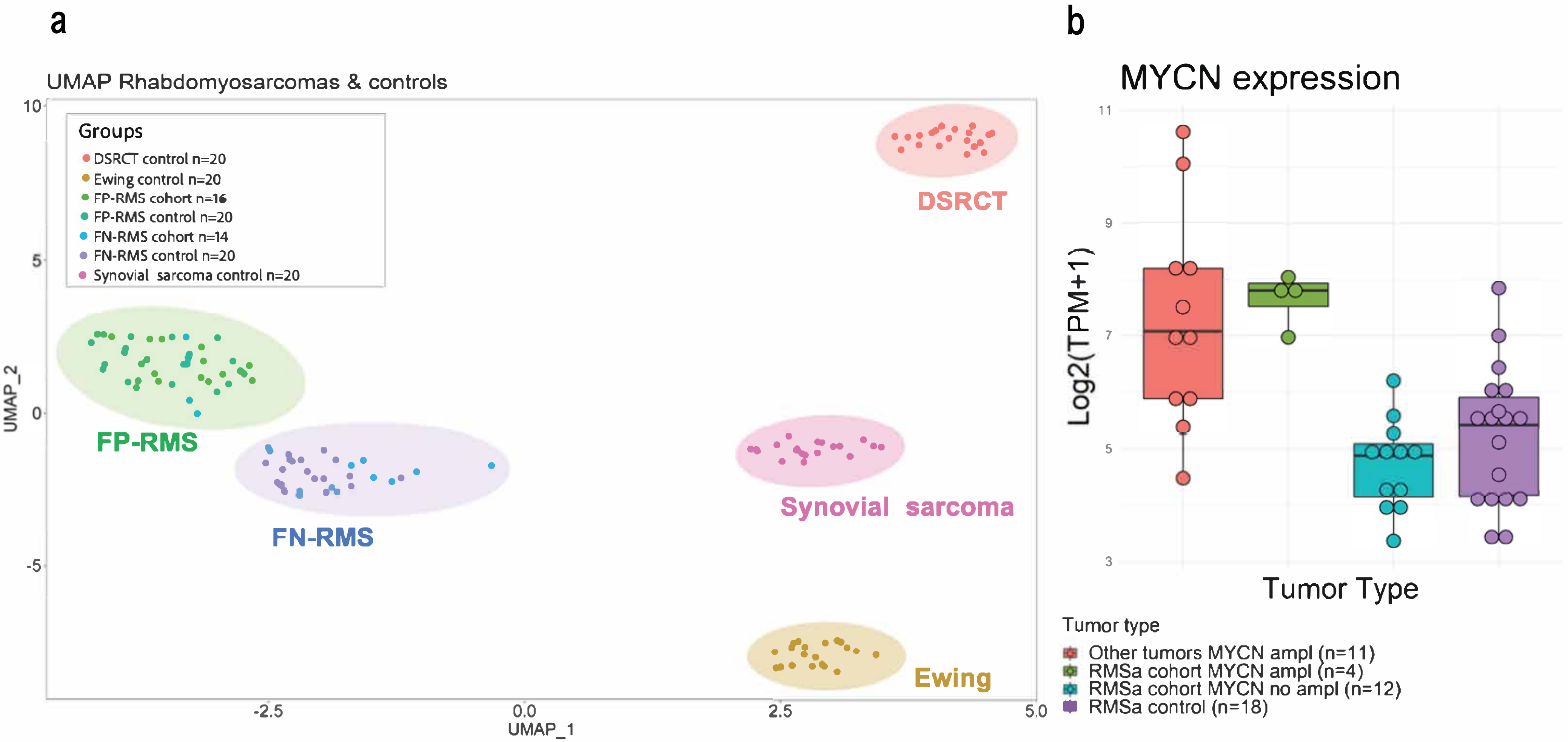

Supplementary Figure 3: a. RNA sequencing unsupervised clustering (UMAP representation) of study group and control groups including other pediatric/young adult sarcomas (Ewing sarcoma, n=18, FP-RMS, n=18, FN-RMS, n=22, desmoplastic small round cell tumors, n=12 and synovial sarcoma, n=18). **b.** Boxplot representation of *MYCN* expression data showing high mRNA levels in the study FP-RMS cohort harboring *MYCN* amplification (n=4) (green) compared to non-amplified FP-RMS sampes (n=17) (red), at similar levels with other tumors with MYCN amplifications. Other FN-RMS and FP-RMS control groups show low level of MYCN expression for comparison. Center line corresponds to the median; lower and upper hinges correspond to 25th and 75th percentiles; upper and lower whiskers correspond to 1.5 x inter-quartile range.

Supplementary Table 1: Gene list included in the high coverage RMS DNA-based targeted custom panel, CHEWIE. Designed for tumor samples and circulating DNA.

AKT1	DICER1	MYCN
AKT2	EZH2	MYOD1
AKT3	FBXW7	NF1
ALK	FGFR3	NRAS
BCOR	FGFR4	PAX3
BRAF	HRAS	PDGFRA
CCND1	IDH1	PDGFRB
CCND2	IDH2	PIK3CA
CDK4	KRAS	PTEN
CDKN2A	MDM2	RB1
CDKN2B	MET	SMARCA2
CTNNB1	MTOR	TP53

Supplementary Table 2. Mutational Signatures of Radiation (RT)-associated Sarcomas

Tumor type	Match to COSMIC signatures	Similarity	Proposed etiology
FP-RMS (primary)	SBS5<br>SBS29	0.788<br>0.9	Unknown (clock-like signature)<br>Tobacco chewing
FP-RMS (primary)	SBS5<br>SBS6<br>SBS29<br>SBS54	0.774<br>0.857<br>0.891<br>0.618	Unknown (clock-like signature)<br>Defective DNA mismatch repair<br>Tobacco chewing<br>Possible sequencing artefact
FN-RMS (primary)	SBS5<br>SBS29<br>SBS6	0.762<br>0.824<br>0.839	Unknown (clock-like signature)<br>Tobacco chewing<br>Defective DNA mismatch repair
FN-RMS (recurrence)	SBS5<br>SBS29<br>SBS6	0.789<br>0.857<br>0.845	Unknown (clock-like signature)<br>Tobacco chewing<br>Defective DNA mismatch repair

FN-RMS: fusion-negative rhabdomyosarcoma; FP-RMS: fusion-positive rhabdomyosarcoma; SBS: Single base substitution.

Supplementary Table 3: ctDNA variant screening and detection based on selected alterations in the tumor samples. All patients selected had at least one alteration found in the tumor that could potentially be detected by our ctDNA platform. In **bold**, alterations found in the primary tumor that could be candidate for ctDNA detection.

	Alterations Detected in Primary Tumor					ctDNA Variant Detection					
Patient ID	Fusion	SNV type	SNV Gene	SNV AR in tumour (%)	Gene level CNV gene and type	Variant detected at diagnosis	Variant detected at relapse	Nr of timepoints # with any variant detected	Nr of Time-points with cancer present	Rate of detection	Vital Status
FP-RMS1	***PAX3::FOXO1* fusion**	none	na	na	***CDKN2A* LOH**	yes	yes	11	11	100%	Deceased
FP-RMS3	***PAX3::FOXO1* fusion**	non-synonsymous	***RB1; PIK3CA***	47; 17	none	yes	yes	9	9	100%	Deceased
FP-RMS4	***PAX3::FOXO1* fusion**	none	na	na	***CDK4* Gain**	yes	yes	4	6	67%	Alive
FP-RMS5	***PAX3::FOXO1* fusion**	none	na	na	***CDK4* Gain; *CDKN2A* Deletion**	yes	yes	3	3	100%	Deceased
FP-RMS6	***PAX3::FOXO1* fusion**	none	na	na	***CDK4* Amplification**	yes	no	2	4	50%	Alive
FP-RMS7	***PAX3::FOXO1* fusion**	Stop gain (snv)	***CDKN2A***	7	***MYCN* Amplification**	yes	yes	4	4	100%	Deceased
FP-RMS8	***PAX3::FOXO1* fusion**	non synonymous	***MYCN***	60	***CDK4* Amplification**	Timepoint not available	yes	6	7	86%	Deceased
FN-RMS3	no	non synonymous	***NRAS; FBXW7***	47; 35	none	Timepoint not available	yes	1	1	100%	Deceased
FN-RMS6	no	Stop gain (SNV), non-synonymous	***BCOR; HRAS***	51; 8	***MDM2* Amplification**	yes	yes	5	6	83%	Deceased
FN-RMS8	no	none	na	na	***BCOR* Deletion**	yes	yes	5	7	71%	Deceased

Supplementary Table 4: Clinical information at diagnosis for patients for whom ctDNA analysis was done.

Patient Code	RISK GROUP	N status	M status	Tumor Localisation	Tumor Size (<5cm = 0, >5cm =1)
FN-RMS3	High	1	0	Abdominopelvis	1
FN-RMS6	High	1	1	Head and Neck	1
FN-RMS8	High	0	1	Head and Neck	2
FP-RMS1	High	1	0	Extremity	1
FP-RMS3	High	1	0	Abdominopelvis	1
FP-RMS4	High	1	1	Extremity	1
FP-RMS5	High	1	0	Extremity	1
FP-RMS6	High	1	1	Extremity	1
FP-RMS7	High	1	1	Extremity	1
FP-RMS8	High	0	0	Head and Neck	0

Supplementary Table 5: Summary of ctDNA detection based on type of alterations. *All timepoints selected at times of clinically assessed active disease; †List of targeted SNVs and CNVs available in Table S1.

Variant type	N patients (total = 10)	N Timepoints*	N positive timepoints	% positive timepoints
PAX3::FOXO1 fusion	7 FP-RMS	40	33	83%
SNV	5 Patients with detectable SNVs†	27	18	67%
CNV	8 Patients with gene level CNVs†	44	29	66%
