# Supplementary material for: Sequential genomic analysis using a multisample/multiplatform approach to better define rhabdomyosarcoma progression and relapse
Source: NPJ Precis Oncol. 2023 Sep 20;7:96. doi: 10.1038/s41698-023-00445-1 (PMC10511463; doi:10.1038/s41698-023-00445-1)
Supplement: Supplementary file 2 — Reporting Summary [file 41698_2023_445_MOESM2_ESM.pdf]

nature portfolio

Corresponding author(s): Cristina Antonescu, Gaelle Pierron

Last updated by author(s): May 25, 2023

Reporting Summary

Nature Portfolio wishes to improve the reproducibility of the work that we publish. This form provides structure for consistency and transparency in reporting. For further information on Nature Portfolio policies, see our Editorial Policies and the Editorial Policy Checklist.

Statistics

For all statistical analyses, confirm that the following items are present in the figure legend, table legend, main text, or Methods section.

n/a	Confirmed	
☐	☒	The exact sample size (*n*) for each experimental group/condition, given as a discrete number and unit of measurement
☐	☒	A statement on whether measurements were taken from distinct samples or whether the same sample was measured repeatedly
☐	☒	The statistical test(s) used AND whether they are one- or two-sided<br>*Only common tests should be described solely by name; describe more complex techniques in the Methods section.*
☐	☒	A description of all covariates tested
☐	☐	A description of any assumptions or corrections, such as tests of normality and adjustment for multiple comparisons
☐	☒	A full description of the statistical parameters including central tendency (e.g. means) or other basic estimates (e.g. regression coefficient) AND variation (e.g. standard deviation) or associated estimates of uncertainty (e.g. confidence intervals)
☐	☒	For null hypothesis testing, the test statistic (e.g. *F*, *t*, *r*) with confidence intervals, effect sizes, degrees of freedom and *P* value noted<br>*Give P values as exact values whenever suitable.*
☒	☐	For Bayesian analysis, information on the choice of priors and Markov chain Monte Carlo settings
☒	☐	For hierarchical and complex designs, identification of the appropriate level for tests and full reporting of outcomes
☒	☐	Estimates of effect sizes (e.g. Cohen's *d*, Pearson's *r*), indicating how they were calculated

Our web collection on statistics for biologists contains articles on many of the points above.

Software and code

Policy information about availability of computer code

Data collection	No software was used for data collection.
Data analysis	MSK-IMPACT Pipeline, ONCO-kb, Bowtie2, Genome Analysis Tool Kit (GATK) (HaplotypeCaller & UnifiedGenotyper) and MuTect2 calling, VarSome 10.1 14 and Alamut Visual Plus V1.4 15, Sequenza (v2.1.0), DNAcopy (v1.52.0), and FREEC (v11.5), Defuse V0.6.2, StarFusion v1.2.0 (STAR v 2.5.4a), Fusion Catcher v1.00, FusionMap (Oshell toolkit v10.0.1.50) and ARRIBA v1.2.0., STAR (v2.5.3a, on hg19 reference genome), Haplotype Caller (GATK v.3.5) and Mutect2 (GATK v.4), ANNOVAR (v2018Apr16), Python package hgvs (v1.2.5), tximport (R library), SAMtools (v1.14), IchorCNA (v0.2.0), WisecondorX (v1.2.4)

For manuscripts utilizing custom algorithms or software that are central to the research but not yet described in published literature, software must be made available to editors and reviewers. We strongly encourage code deposition in a community repository (e.g. GitHub). See the Nature Portfolio guidelines for submitting code & software for further information.

Data

Policy information about availability of data

All manuscripts must include a data availability statement. This statement should provide the following information, where applicable:

- Accession codes, unique identifiers, or web links for publicly available datasets
- A description of any restrictions on data availability
- For clinical datasets or third party data, please ensure that the statement adheres to our policy

The anonymized MSKCC variant level data (mutation, copy number, structural variants including fusion) are available at: https://cbioportal.mskcc.org/study/summary?id=soft_tissue_msk_2023. The anonymized Institut Curie variant level data (mutation, copy number, structural variants including fusion for tumors and ctDNA) will be publicly available before publication on the EGA European Genome-Phenome Archive repository.

Research involving human participants, their data, or biological material

Policy information about studies with human participants or human data. See also policy information about sex, gender (identity/presentation), and sexual orientation and race, ethnicity and racism.

Reporting on sex and gender	Our findings apply to sex and not to gender. No gender analysis were done due to the lack of such information in our retrospective cohort.
Reporting on race, ethnicity, or other socially relevant groupings	No race, ethnicity or socially relevant groupings were used to discriminate sub-populations within this collection of patients. Therefore no analysis on such parameters have been made available.
Population characteristics	The population within our study is a majority of pediatric patients and adolescents/young adults (ratio 1:1) with rhabdomyosarcoma (both embryonnal and alveolar). Most cases have no previous medical history. The sex ratio is 1:1.
Recruitment	We included patients having a confirmed pathologic and molecular diagnosis of metastatic/relapsed RMS between 2003 - 2022 for whom sequential NGS data was available, in most cases including primary and relapse samples. Ten cases underwent molecular analysis as part of the European MAPPYACTS trial8. All FP-RMS samples were confirmed for the presence of FOXO1 fusion either by RNAseq (IC) or Archer FusionPlex (MSKCC)9. A total of 35 patients were selected from both institutions (20 from MSKCC, 15 from IC), which included both pediatric and young adults. Pathology reports and clinical charts were reviewed for the tumor size, treatment information, date and location of metastatic disease or relapse, last follow-up and status of disease. The major selection bias being the availability of the material and/or molecular and clinical data.
Ethics oversight	The study was approved by the Institutional Review Board (IRB) committees at both institutions (Institut Curie, Paris and MSKCC, NY) and all participants provided written informed consent to take part in the study.

Note that full information on the approval of the study protocol must also be provided in the manuscript.

Field-specific reporting

Please select the one below that is the best fit for your research. If you are not sure, read the appropriate sections before making your selection.

☒ Life sciences ☐ Behavioural & social sciences ☐ Ecological, evolutionary & environmental sciences

For a reference copy of the document with all sections, see nature.com/documents/nr-reporting-summary-flat.pdf

Life sciences study design

All studies must disclose on these points even when the disclosure is negative.

Sample size	35 patients, 72 tumors, 108 peripheral DNA samples (blood or bone marrow). Patients were selected based on availability of their material and no calculations of required sample size were conducted given the context of rare disease.
Data exclusions	No exclusion criteria - only non-inclusion.
Replication	Results were identical on both platforms from two different institutions.
Randomization	No randomization process was used in this study.
Blinding	Data is provided as raw as possible and no blinding is therefore required given the descriptive and retrospective nature of our study.

Reporting for specific materials, systems and methods

We require information from authors about some types of materials, experimental systems and methods used in many studies. Here, indicate whether each material, system or method listed is relevant to your study. If you are not sure if a list item applies to your research, read the appropriate section before selecting a response.

Materials & experimental systems

n/a	Involved in the study
☒	☐ Antibodies
☒	☐ Eukaryotic cell lines
☒	☐ Palaeontology and archaeology
☒	☐ Animals and other organisms
☐	☒ Clinical data
☒	☐ Dual use research of concern
☒	☐ Plants

Methods

n/a	Involved in the study
☒	☐ ChIP-seq
☒	☐ Flow cytometry
☒	☐ MRI-based neuroimaging

Clinical data

Policy information about clinical studies

All manuscripts should comply with the ICMJE guidelines for publication of clinical research and a completed CONSORT checklist must be included with all submissions.

Clinical trial registration	EpSSG RMS 2005 STS 2006 04, D9802, D9803, ARST0331, ARST0431, ARST0531. Patients were registered from multiple different clinical trials. https://clinicaltrials.gov/ct2/show/NCT00354835 ; https://clinicaltrials.gov/ct2/show/NCT00354744 ; https://clinicaltrials.gov/ct2/show/NCT00075582
Study protocol	Not a study but a retrospective collection. Inclusion criteria described above.
Data collection	Data collection was done between April 2022 and August 2023.
Outcomes	Primary outcome was any somatic molecular event, secondary outcome was death. Kaplan-Meier curves were compared using the logrank test. Cox proportional hazards regression was used to identify prognostic factors for overall survival and event-free survival.
